# Regional Values of Diffusional Kurtosis Estimates in the Healthy Brain

**DOI:** 10.1002/jmri.23857

**Published:** 2012-10-10

**Authors:** Jimmy Lätt, Markus Nilsson, Ronnie Wirestam, Freddy Ståhlberg, Nils Karlsson, Mikael Johansson, Pia C Sundgren, Danielle van Westen

**Affiliations:** 1 Center for Medical Imaging and Physiology, Skåne University HospitalLund, Sweden; 2 Lund University Bioimaging Center, Lund UniversitySweden; 3 Department of Medical Radiation Physics, Lund UniversityLund, Sweden; 4 Department of Diagnostic Radiology, Lund UniversityLund, Sweden; 5 Department of Psychology, Lund UniversityLund, Sweden

**Keywords:** brain, diffusion, kurtosis

## Abstract

**Purpose:**

To provide estimates of the diffusional kurtosis in the healthy brain in anatomically defined areas and list these along previously reported values in pathologies.

**Materials and Methods:**

Thirty-six volunteers (mean age = 33.1 years; range, 19–64 years) underwent diffusional kurtosis imaging. Mean kurtosis (MK), radial kurtosis (RK), mean diffusivity (MD), radial diffusivity (RD), and fractional anisotropy (FA) were determined in 26 anatomical structures. Parameter estimates were assessed regarding age dependence.

**Results:**

MK varied from 1.38 in the splenium of the corpus callosum to 0.66 in the caudate head, MD varied from 0.68 to 0.62 μm^2^/ms and FA from 0.87 to 0.29. MK, and FA showed a strong positive correlation, RK and RD a strong negative correlation. Parameter estimates showed age correlation in some regions; also the average MK and RK for all WM and all GM areas, respectively, were negatively correlated with age.

**Conclusion:**

DKI parameter estimates MK and RK varied depending on the anatomical region and varied with age in pooled WM and GM data. MK estimates in the internal capsule, corpus callosum, and thalamus were consistent with previous studies. The range of values of MK and RK in healthy brain overlapped with that in pathologies. J. Magn. Reson. Imaging 2013;37:610–618. © 2012 Wiley Periodicals, Inc.

DIFFUSIONAL KURTOSIS IMAGING (DKI) is an extension of diffusion tensor imaging (DTI) and aims at describing the non-Gaussian aspect of water diffusion (1). The mean kurtosis (MK) is a dimensionless parameter that reflects the degree of diffusion restriction and the radial (RK) is its perpendicular component. MK is regarded an index of the complexity of tissue microstructure such as the density, orientation, and degree of organization of cell membranes, axon sheaths, and myelin layers. Accordingly, parameters derived from DKI are highly sensitive to changes in the microstructural tissue organization occurring during postnatal maturation of the normal brain, and, in fact, more so than conventional DTI parameters (2). Thus, DKI metrics are potential markers for both normal development and disease.

Before application of DKI as a clinical tool, knowledge of regional DKI values in the healthy brain is essential. Previous reports on DTI metrics have shown little variation in mean diffusivity (MD) across anatomical structures, but considerable variation in fractional anisotropy (FA). For example, in 30-year-old healthy individuals MD was approximately 0.7 μm^2^/ms in the putamen and 0.72 μm^2^/ms in the posterior limb of the internal capsule (PLIC) while FA was 0.2 and 0.72, respectively (3). The relative anisotropy (RA), a metric closely related to FA, varies up to 50% within the internal capsule alone (4). Until now, the scarce reports on regional values of the kurtosis in the healthy human brain include measurements in a limited number of anatomical locations, suggesting that MK varies depending on anatomical location (5–8).

In addition, the human brain is subject to structural and morphological changes during development and ageing with increases in white matter (WM) volume occurring during adolescence and early adulthood, and subsequent decreases with ageing (3, 9, 10). GM has been shown to decline nonlinearly in density between the ages of 7 and 60 years with the rate depending on the location (10). Age-dependent changes in DTI metrics have been reported extensively (11–14) with FA and MD in white matter tracts changing over time, consistent with the abovementioned alterations in tissue microstructure (15). Age dependency in healthy brain has also been reported for DKI metrics (6, 8, 16). Helpern et al found a significant kurtosis increase from 12 to 18 years of age, particularly in the radial direction, in a large region of interest (ROI) covering the frontal part of the brain (6). Falangola et al found a decrease in the peak histogram location for mean kurtosis in white matter from 60 to 80 years (8). Lu et al found a gradual decrease in gray-white contrast histogram peaks from young controls (n = 5; age, 35.0 ± 6.0) to elderly controls (n = 6; age, 70.0 ± 9.5).

The purpose of the present study was to determine MK and RK as well as MD and FA, in a large number of anatomically defined regions in the healthy brain, including WM areas as well as deep nuclei, in individuals aged between 20 and 65 years. Our primary motivation was that if MK is to become a useful aid in early diagnosis and characterization of disease by contributing information on microstructure, values of MK obtained in pathologies such as intracranial tumors or stroke lesions must be interpreted in relation to those obtained in healthy controls. Thus, in this work, we present a reference material with ROI-based values of DKI metrics from a large number of anatomically defined areas that may be used for comparison. Because DKI metrics have previously been shown to vary with age in some regions, we also probed for age dependence. Lastly, we present values of MK in pathologies as previously reported in the literature.

## MATERIAL AND METHODS

### Subjects

Thirty-six self-reported neurologically healthy individuals (16 males and 20 females) between 20 and 64 years old (mean age of all subjects: 33.1 years, standard deviation 12.5 years; mean age for women: 38. 4 years, standard deviation 12.4 years; mean age for men: 35.6 years, standard deviation 10.6 years) were recruited to the study. Apart from minor WM hyperintensities in some of the older individuals, no signal changes were present on FLAIR images. The study was approved by the local ethics board and all subjects gave their written informed consent before inclusion in the study.

### Data Acquisition

Images were acquired using a Philips Achieva 3 Tesla (T) scanner (Philips Medical Systems, Best, The Netherlands), equipped with an eight-channel head coil. DKI image acquisition was performed using a pulsed-gradient spin echo (PGSE) sequence with echo-planar imaging (EPI) readout (SENSE factor = 2.0, TR/TE = 5700 ms/76 ms, Δ/δ = 37.4 ms/17.9 ms, 5400 ms/76 ms, partial Fourier=75%, field of view 256 × 256 mm^2^, reconstructed image resolution 2 × 2 × 2 mm^3^). Diffusion encoding (without averaging) was applied i n 15 directions with b-values 0, 500, 1000, 2500, and 2750 ms/μm^2^, giving a total scan time of 6 min. The employed diffusion encoding gradients (x, y, z), given in the reference of the image volume were: -0.219, -0.899,-0.379; 0.388,-0.147, 0.909; 0.803, 0.342, -0.487; -0.852, 0.443, 0.234; -0.235, 0.727,-0.645; 0.729, 0.681, 0.071; -0.581, 0.177, 0.794; -0.989, -0.082,-0.116; 0.373,-0.748,-0.549; 0.051, 0.400, 0.915; -0.305,-0.885, 0.352; 0.787,-0.435, 0.438; -0.259,-0.369, 0.893; 0.672, 0.366, 0.646; 0.371, -0.927, 0.059.

A 5.4-cm-thick slab of 27 slices, was obtained, covering the brain from below the cerebral peduncles to superior to the hand area of the primary motor cortex; because our long-term goal was to apply diffusion kurtosis imaging in the clinic, we accepted incomplete coverage of the brain to achieve clinically reasonable scan times, In addition, transversal FLAIR images (TR = 12,000 ms, TE = 140 ms, TI = 2500 ms) were acquired to rule out pathology.

### Data Analysis

**Figure 1:**
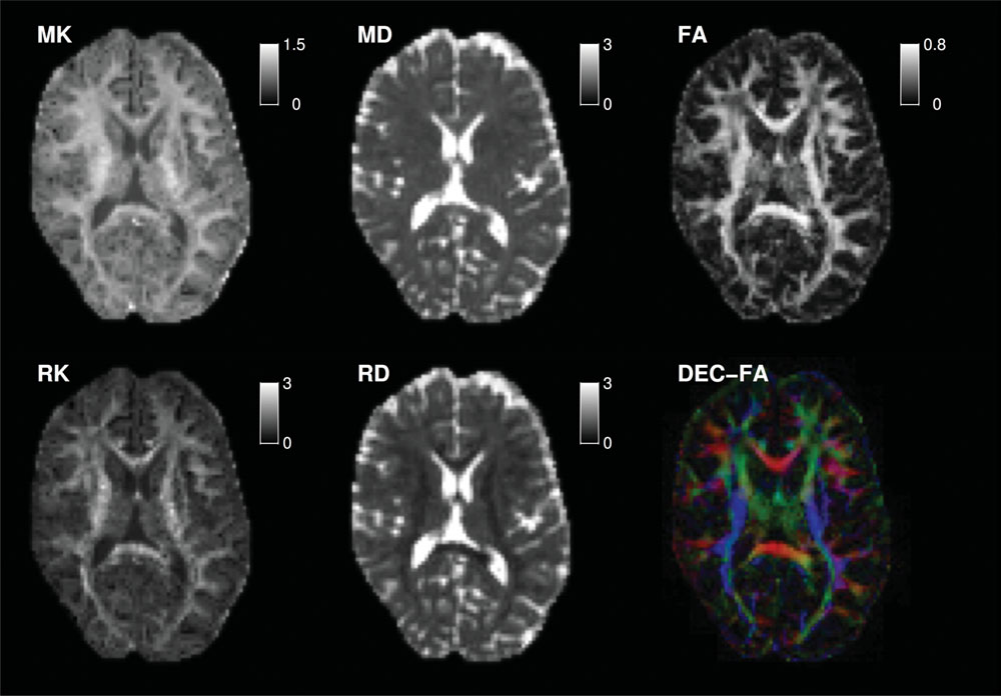
Parameter maps of MK, RK, MD, RD, FA, and directionally encoded FA (DEC-FA) from one participant, a 34-year-old healthy male. The contrast in the MK and RK maps is similar to that in the FA map with high values in WM and low values in GM.

The diffusion-weighted volumes were geometrically corrected for subject motion and eddy currents using the affine registration algorithm (FLIRT) provided in the ElastiX package (http://elastix.isi.uu.nl). The diffusion kurtosis model, described previously (1, 7, 17, 18), was fitted to the diffusion-weighted signal intensities in each voxel by nonlinear least squares minimization using Matlab (The MathWorks, Natick, MA). The fitted kurtosis tensor was used to calculate MK and RK as described by Tabesh et al (19). The FA, MD and RD were calculated from the diffusion tensor in the diffusion kurtosis model using conventional equations (19). Finally, parametric maps were constructed for each parameter (Fig. 1). Although values of the axial kurtosis (AK), that is the kurtosis parallel to the WM tracts (1), can be determined from our data, we chose not to report these in the present work, because estimation of AK is highly susceptible to bias resulting from the non-Gaussian noise distribution.

### Regional Values

**Figure 2:**
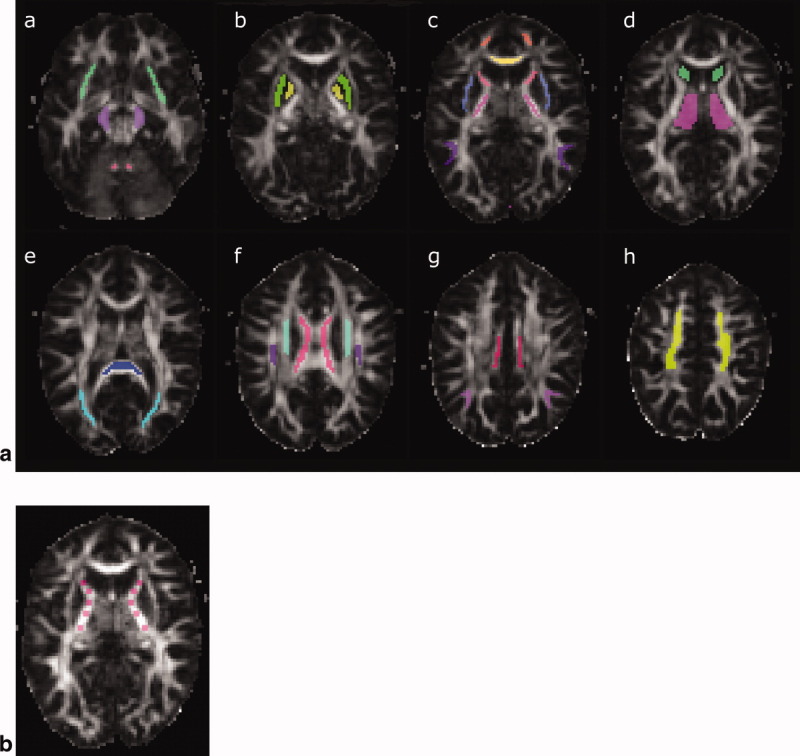
**a**: ROIs for measurement in the anatomical areas reported in Table 1, overlaid on the FA-map from the same individual as in Figure 1, (a) in the IFO (green), the CST at the level of the cerebral peduncle (purple) and the temporal cingulate (pink); in the putamen (green) and pallidum (yellow) (**b**); in the ALIC (red), PLIC (pink), external capsule (light purple), genu of the corpus callosum (CC) (yellow) and the frontal (orange) and temporal (purple) subcortical white matter (**c**); in the caudate head (green) and thalamus (purple) (**d**); in the splenium of the CC (dark blue) and the posterior IFL (light blue) (**e**); in the body of the CC (pink), the posterior SLF (purple), and the corona radiate (light blue) (**f**); in the cingulate (red) and the parietal subcortical white matter (purple) (**g**); and in the centrum semiovale (green) (**h**). **b**: ROIs for measurement anteroposteriorly along the internal capsule, the so-called gradient, (i) anteriorly in its anterior limb (ALIC), (ii) in its middle segment, (iii) in the genu, (iv) in middle segment of its posterior limb (PLIC), and (v) posteriorly in the PLIC.

Directionally encoded (DEC) FA maps were inspected and regions of interest (ROIs) that were reliably identifiable in all subjects, and in which partial volume effects could be minimized, were included in the study. With regard to the latter issue, special attention was given to areas close to cerebrospinal fluid (CSF) to avoid contamination of any ROI with freely diffusing CSF. All ROIs were placed using TrackVis version 0.5.1 (http://trackvis.org/). First, 22 anatomically defined structures were delineated in one slice representative for the region in question (Table 1, Fig. 2a). Second, five 2 × 2 voxel ROIs were placed in the internal capsule (IC), (i) anteriorly in its anterior limb (ALIC), (ii) in its middle segment, (iii) in the genu, (iv) in middle segment of its posterior limb (PLIC), and (v) posteriorly in the PLIC (Fig. 2b). Previously a gradual increase in FA has been reported along this so-called “gradient” in the IC (4, 20). Third, MK in the cerebrospinal fluid (CSF) occupying the lateral ventricles was determined in a subsample of ten individuals, using four ROIs of 10–25 voxels. MK in free water is of interest because it has been reported to be as high as 0.4 in CSF (8), although its value in freely diffusing water theoretically is zero (1).

**Table 1:** Regional Values, Mean and Standard Deviation, and Pearson Correlation With Age*

ROI	MK	RK	MD [μm^2^/ms]	RD [mm^2^/s]	FA
External capsule	0.81 ± 0.05*^/-^	1.02 ± 0.09	0.90 ± 0.05	0.70 ± 0.04	0.41 ± 0.03
ALIC	1.04 ± 0.10	1.60 ± 0.28	0.87 ± 0.05	0.53 ± 0.05	0.60 ± 0.04
PLIC	1.23 ± 0.09	2.04 ± 0.23	0.89 ± 0.09	0.45 ± 0.07	0.71 ± 0.04
CC, body	1.17 ± 0.07	2.54 ± 0.34	0.92 ± 0.07	0.38 ± 0.07	0.78 ± 0.04
CC, genu	1.06 ± 0.11*^/-^	2.07 ± 0.45^†/-^	0.93 ± 0.06	0.36 ± 0.07	0.80 ± 0.04
CC, splenium	1.32 ± 0.09	2.72 ± 0.41^†/-^	0.89 ± 0.09	0.31 ± 0.07*^/-^	0.83 ± 0.03†^/+^
Centrum semiovale	1.09 ± 0.04*^/-^	1.72 ± 0.16^†/-^	0.80 ± 0.04	0.47 ± 0.05	0.63 ± 0.04
Cingulate, body	1.07 ± 0.07	1.85 ± 0.26*^/-^	0.86 ± 0.07	0.48 ± 0.08	0.66 ± 0.06
Cingulate, temporal	0.85 ± 0.08	1.13 ± 0.21	0.92 ± 0.12	0.60 ± 0.10	0.55 ± 0.05
Corona radiata	1.09 ± 0.04^†/-^	1.49 ± 0.09*^/-^	0.84 ± 0.05	0.56 ± 0.04	0.53 ± 0.03
CST, cerebral crus	1.23 ± 0.07	2.04 ± 0.28	0.88 ± 0.08	0.40 ± 0.09	0.75 ± 0.05
IFO, anterior basal	0.86 ± 0.07^†/-^	1.29 ± 0.19^†/-^	0.89 ± 0.05^†/-^	0.58 ± 0.05	0.54 ± 0.05
ILF, posterior	0.96 ± 0.06*^/-^	1.60 ± 0.18	0.90 ± 0.06	0.51 ± 0.07	0.64 ± 0.05
SLF, posterior	1.11 ± 0.04	1.84 ± 0.13	0.83 ± 0.04	0.50 ± 0.05	0.62 ± 0.04
Frontal sWM	0.94 ± 0.05^†/-^	1.23 ± 0.12*^/-^	0.91 ± 0.05	0.66 ± 0.05	0.48 ± 0.04^†/-^
Parietal sWM	1.00 ± 0.05	1.41 ± 0.12	0.86 ± 0.06	0.56 ± 0.07	0.56 ± 0.05
Temporal sWM	0.96 ± 0.07	1.27 ± 0.13	0.88 ± 0.08*^/-^	0.61 ± 0.06	0.52 ± 0.03
Caudate head	0.61 ± 0.08	0.59 ± 0.07	0.87 ± 0.05	0.80 ± 0.04	0.14 ± 0.03
Globus pallidus	1.06 ± 0.08	1.05 ± 0.10*^/-^	0.86 ± 0.08	0.74 ± 0.06	0.27 ± 0.04
Putamen	0.67 ± 0.08	0.61 ± 0.08	0.79 ± 0.03*^/-^	0.73 ± 0.03^†/-^	0.15 ± 0.02
Thalamus	0.86 ± 0.07*^/-^	0.92 ± 0.09*^/-^	0.87 ± 0.10	0.73 ± 0.09	0.32 ± 0.03
IC, gradient 1	0.89 ± 0.10	1.32 ± 0.26	0.85 ± 0.06*^/+^	0.59 ± 0.06†^/+^	0.48 ± 0.06
IC, gradient 2	1.05 ± 0.12	1.58 ± 0.30	0.90 ± 0.07	0.56 ± 0.08	0.60 ± 0.05
IC, gradient 3	1.10 ± 0.10	1.51 ± 0.22	0.92 ± 0.09	0.59 ± 0.08	0.57 ± 0.05
IC, gradient 4	1.21 ± 0.09	1.88 ± 0.24	0.91 ± 0.12	0.51 ± 0.11	0.65 ± 0.05
IC, gradient 5	1.21 ± 0.11	2.02 ± 0.31	0.87 ± 0.08	0.41 ± 0.07	0.74 ± 0.04

* An asterisk indicates *P* < 0.05, and a dagger (^†^) indicates *P* < 0.01.

Positive correlations are indicated by a plus sign, negative by a minus sign.

Except for the genu and splenium of the corpus callosum, ROIs were placed bilaterally. White matter (WM) ROIs were defined on FA maps and color-coded FA maps. The slice containing the voxels with the highest FA was selected for delineation of any structure and the full length of the structure in the selected slice was included. Voxels adjacent to neighboring structures were excluded to avoid partial volume effects. Gray matter (GM) ROIs were defined using FA maps and images acquired without diffusion weighting. All ROIs were placed in the transverse plane on noninterpolated, nonsmoothed images and without access to kurtosis maps. For each ROI, the average value of each parameter estimate was extracted. Next, the mean values of the right and left ROIs of bilateral anatomical structures were averaged to provide a single value for each bilateral structure. Values presented for singular structures were derived from a single ROI average.

### Statistical Analysis

Statistical analysis was performed using Matlab. For each parameter and area, age-dependency was probed for using Pearson's correlation. DTI parameters previously have shown nonlinear correlation with age (3, 15), therefore also quadratic correlation with age was probed for; the quadratic fit was compared with the linear fit (*P* < 0.01) using an *F*-test, with F calculated according to:


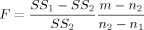


where *SS*_1_ and *SS*_2_ are the sum-of-squares of the residuals between the data and the linear and quadratic fits, m is the number of data points (m = 36), *n*_2_ = 3 and *n*_1_ = 2.

## RESULTS

### Parametric Maps

Parametric maps from one representative individual, a 34-year-old male, are shown in Figure 1. The contrast in the MK and RK maps was similar to that in the FA maps, with high parametric values in WM and lower values in GM; however, there was somewhat more contrast on FA maps between WM and GM compared with that available in the MK map with GM values being closer to nil for FA than for MK. The contrast on MK and RK maps was similar, because RK has a large impact on MK, similar to the impact of RD on MD. MK and FA share the theoretical property of having an expected value of zero in regions where the water diffusion is free and unrestricted, such as in the ventricles.

### Regional Values of DKI Metrics

Regional values of obtained DKI parameters are presented in Table 1. MK varied from 0.61 ± 0.08 in the caudate head to 1.32 ± 0.09 in the splenium of the corpus callosum, while RK in these locations varied from 0.59 ± 0.07 to 2.72 ± 0.41, MD from 0.87 ± 0.05 μm^2^/ms to 0.89 ± 0.09 μm^2^/ms, RD from 0.80 ± 0.04 μm^2^/ms to 0.31 ± 0.07 μm^2^/ms and FA from 0.14 ± 0.03 to 0.83 ± 0.03. In the internal capsule values of MK most anteriorly and most posteriorly were 0.89 ± 0.10 and 1.21 ± 0.11, respectively, while RK was 1.32 ± 0.26 and 2.02 ± 0.31, MD 0.85 ± 0.06 and 0.87 ± 0.08 μm^2^/ms, RD 0.59 ± 0.06 and 0.41 ± 0.07 and FA 0.48 ± 0.06 and 0.74 ± 0.04 in these locations. The value of MK in the CSF occupying the lateral ventricles was 0.35 ± 0.03 (n = 10).

**Figure 3:**
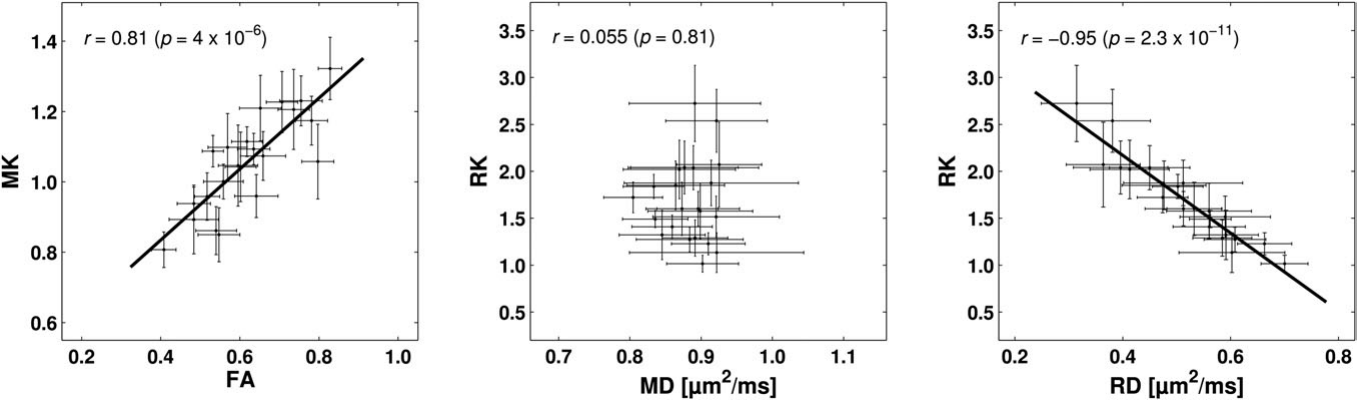
Correlation between DKI metrics in white matter, means and standard deviations; a strong positive correlation was found between MK and FA and a strong negative correlation between RK and RD, while RK and MD did not correlate.

A strong positive correlation between MK and FA was observed in white matter (Fig. 3); the correlation coefficient was *r* = 0.81 (*P* < 10^−5^). RK correlated negatively with RD, with *r* = −0.95 (*P* < 10^−10^), but not with MD. The coefficient of variation, defined as the ratio of the standard deviation across all areas (except the IC gradient) to the corresponding average value, was 0.18, 0.37, 0.04, 0.25, and 0.36 for MK, RK, MD, RD, and FA, respectively. This showed that RK and FA varied the most across the structures, while nearly no variation was found in MD.

### Age Dependence of DKI Metrics

**Figure 4:**
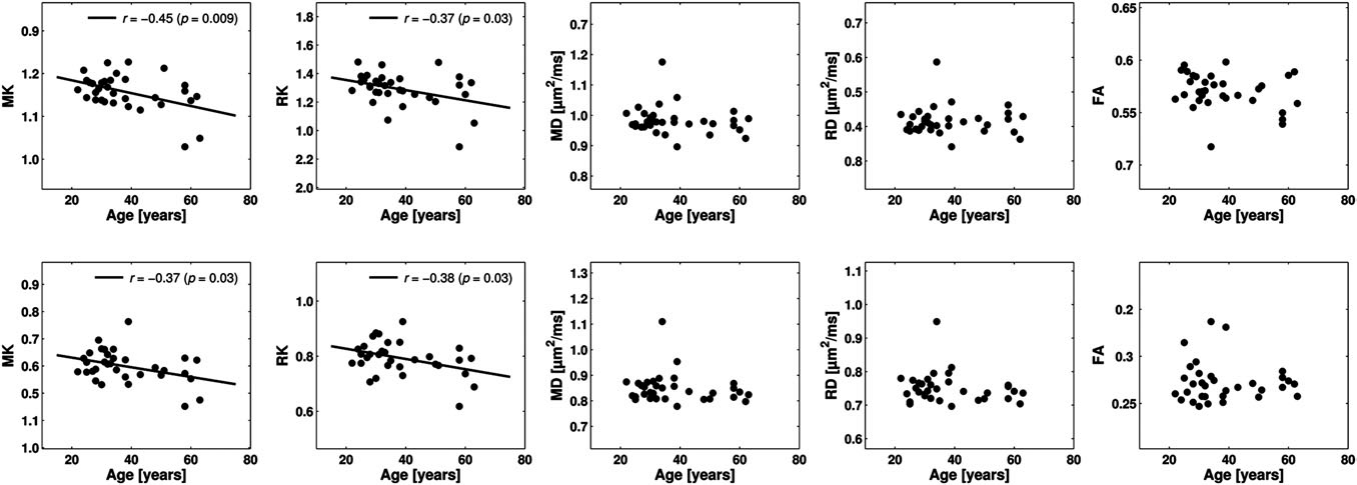
Age dependence of DKI metrics, average from all WM areas (upper panel) and all GM structures (lower panel), Pearson's correlation. MK and RK correlate negatively with age in GM and WM; MD, RD, and FA did not show age dependence.

Age dependence was found for MK and RK in several areas with *P* < 0.01, namely for MK in the corona radiata, IFO, and frontal SWM, and with *P* < 0.05, in the capsula externa, genu of the corpus callosum, centrum semiovale, ILF, and the thalamus. In all structures, MK declined with age. For RK, age dependence was found with *P* < 0.01 in the body and splenium of the corpus callosum, centrum semiovale, and IFO, and with *P* < 0.05 in the body of the cingulum, corona radiate, frontal SWM, globus pallidus, and in the thalamus (Table 1). Again, RK declined with age in all structures. Some areas showed age dependence for MD, RD, and FA (Table 1). Figure 4 shows graphical representations of the investigated parameters versus age for the average of all white and gray matter structures investigated. Significant correlation with age and was found only for MK and RK, in both white and gray matter (*P* < 0.05).

## DISCUSSION

Diffusional kurtosis imaging (DKI) is an extension of diffusion tensor imaging (DTI), proposed for characterization of the non-Gaussian random motion of water molecules (1). Qualitatively, the kurtosis is a measure of the heterogeneity of the diffusion environment, with presumed increased sensitivity for pathology and therefore possibly an early marker of disease, for instance in Parkinson disease (21). In this work, we present a reference material with values of DKI metrics in a large number of anatomically defined areas (Table 1). Because DKI is an extension of DTI, it is reasonable to assume that DKI is potentially useful for many of the same applications as DTI, such as stroke, tumor, and neurodegenerative disease. In this respect, knowledge on regional values of the kurtosis in healthy brain tissue is supposedly useful for comparison with corresponding measures from pathologies. We have searched the literature for values of diffusion kurtosis estimates in disease and found that quantitative measures of DKI parameters from various pathologies have not been extensively reported. Table 2 provides an overview of those works in the literature that include values of parameter estimates; otherwise, reports may for example include histograms of mean kurtosis in WM or GM (6, 8, 16). When comparing values of DKI parameter estimates in healthy brain to those reported in pathologies, interestingly values from intracranial tumors (0.46 in grade II, 0.62 in grade III, 0.81 in grade IV gliomas (22) and 0.47 in low grade gliomas, supposedly grade I and II, and 0.60 in high grade gliomas, supposedly grade III and IV) (23) and stroke (1.33 in WM and 0.66 in GM) (24) overlap with those from healthy tissue (0.66 in the caudate head and 1.38 in the splenium of the corpus callosum; Table 1). This suggests that a single measurement on an individual basis may not allow for differentiation between healthy and diseased tissue without comparison with the range of the parameter estimate in a specific region.

**Table 2:** Regional Values in Healthy Brain and in Pathologies From the Literature, With *N* Denoting the Number of Participants

Region	Reference	*N* =	DKI imaging parameters	Voxel size	MK	RK	MD [μm^2^/ms]	RD [μm^2^/ms]	FA
Healthy brain									
Internal capsule	[5]	*20*	32 directions, 2 b-values (1000, 2000 ms/μm^2^)	2 × 2 × 3 mm^3^	1.21 ± 0.04	1.86 ± 0.10	0.84 ± 0.03	0.51 ± 0.04	0.60 ± 0.03
	[1]	*4*	15 directions 6 b-values (0, 500, 1000, 1500, 2000, 2500 ms/μm^2^)	2 × 2 × 2 mm^3^	1.05 ± 0.08		0.84 ± 0.03		
Corpus callosum	[5]	*20*			1.14 ± 0.05	2.25 ± 0.23	0.95 ± 0.03	0.47 ± 0.03	0.70 ± 0.02
Thalamus	[5]	*20*			0.82 ± 0.03	0.93 ± 0.04	0.86 ± 0.03	0.76 ± 0.03	0.24 ± 0.02
	[1]	*4*			0.93 ± 0.03		0.92 ± 0.13		
	[7]	*4*	30 directions 6 b-values (0, 500, 1000, 1500, 2000, 2500 ms/μm^2^)	2.5 × 2.5 × 2.5 mm^3^	0.84 ± 0.02		0.95 ± 0.03		0.27 ± 0.02
Putamen	[5]	*20*			0.79 ± 0.04	0.83 ± 0.6	0.81 ± 0.04	0.76 ± 0.03	0.12 ± 0.01
	[29]	*30*	3 directions, *41 b*-values (0 to 4000 ms/μm^2^ in 100 ms/μm^2^ steps)	2 × 2 × 5 mm^3^	0.71				
Pathologies									
Glioma grade II	[20]				0.48 ± 0.02		1.67 ± 0.2		0.12 ± 0.01
	[30], LGG	*11*	*b* values 700, 1000, and 2800 ms/μm^2^, in 25, 40 and 75 directions, respectively	2.5 × 2.5 × 2.5 mm^3^	0.47	0.49	1.53		0.13
Glioma grade III	[20]	*13*			0.62 ± 0.03		1.46 ± 0.07		0.14 ± 0.01
Glioma grade IV	[20]	*16*			0.81 ± 0.04		1.11 ± 0.06		0.20 ± 0.03
	[30], HGG	*17*			0.60	0.65	1.43		0.15
Subacute ischemia	[21], WM	*1*	15 directions	2 × 2 × 3 mm^3^	1.33		0.33		
	GM		16 *b*-values with *b*_max_ = 7500 s/mm^2^		0.66		0.52		
Putamen (Parkinson)	[29]	*30*			0.93 ± 0.15				

A strong positive correlation between MK and FA was established in white matter and an equally strong negative correlation between RK and RD (Fig. 3). To understand the correlation between DKI parameters, a simple model described by Fieremans et al may be considered (25). The model incorporates water diffusing in two nonexchanging compartments, representing intra-axonal and extracellular water. Following the definition of the kurtosis tensor, the model suggests that RK = 3 AWF/(1 – AWF), where AWF is the axonal water fraction; and conversely that RD = (1 – AWF) RD_h_ with RD_h_ representing the radial diffusivity in the hindered compartment. Although simplistic, this model predicts that a reduction in RK is accompanied by an increase in RD. The model also predicts a correlation between FA and MK, although the predicted relation between these parameters is more complicated. The implication of these correlations is that DKI may not add significant value over DTI in the characterization of normal white matter where DKI and DTI both yield information on the axonal water fraction. In pathology involving, for example, invading tumor cells, DKI may capture information not available to DTI as suggested by Raab et al (22) as well as by Van Cauter et al (23).

Age dependence was probed for, partly to gain more knowledge on DKI and partly because age-related changes may confound clinical data related to the parameter range in a specific region. Previous reports on WM changes during ageing based on DTI measurements, include nonlinear increase of the FA from adulthood onward with white matter gain continuing into approximately 33 years of age followed by nonlinear loss, according to a standard parabola. The diffusivity in WM shows the opposite pattern with a nonlinear decreases until 38 years of age followed by a similar nonlinear increase, i.e., an inverted parabola (15, 26). In GM, FA has been shown to follow an inverted parabola with a minimum at approximately 33 years of age (15, 26); this decrease in FA has been reproduced in other works for the caudate (27) and putamen (12) with results in the latter based on voxel-based analysis. The diffusivity in GM does not correlate with age beyond 20–30 years; in childhood before this, there is a rapid decrease (15, 26). Here, we probed for both linear and quadratic correlation with age and found several regions in which the DKI metrics MK and RK showed significant linear correlation with age. We chose to report the analysis without correcting for multiple comparisons, because a Bonferroni correction for multiple comparisons would yield a significance level of 0.05/130 = 3.8 × 10^−3^, at which no correlation was significant; however, such a low significance level also induces false negative results. Regarding false positives, 130 independent tests on uncorrelated data as performed in the present study may yield 2 to 12 false positives at the 5% level (95% confidence interval of the binomial distribution with *N* = 130, *P* = 0.05) and between 0 and 4 at the 1% level. In comparison, we found 26 and 12 significant correlations at the 5% and 1% level, respectively. Note, we performed tests on dependent data, and thus the predicted number of false positives is probably overestimated, further strengthening the validity of our results. We found a negative correlation with age for MK in WM, where the average MK for all structures ranged from 1.08 at age 20 to 1.02 at age 60 as well as in GM, where the average MK for all structures ranged from 0.83 at age 20 to 0.76 at age 60; similarly the average RK in WM ranged from 1.72 to 1.61, and in GM from 0.83 to 0.75, respectively. These results suggest that our study is underpowered; for a standard deviation of 0.1 in MK, the group sizes required for detecting a difference of 0.06 with a power of 0.8 is 44, i.e., in total 88 subjects being either 20 or 60 years old. This might partly explain why the present work did not entirely reproduce the results from previous studies on age dependency of MD and FA. However, it should be noted that the values of MD and FA obtained in DKI are not directly comparable to those obtained in DTI as the non-monoexponential component of diffusion data that influences the MD and FA obtained from DTI, is instead captured by the kurtosis tensor in DKI. Two previous reports on age dependency for MK are based on histogram analysis; peaks corresponding to all WM and GM have shown a reduction from young controls to elderly controls (8, 16), while one work reported values from a large frontal ROI, including WM, GM, and CSF, in adolescents aged 12–18 years (6). In addition, increases in MK during maturation from birth has been shown in rodents (2), however in this case it may be presumed that changes in tissue microstructure occur at a much faster rate and are of greater magnitude than in the age groups investigated in the present study. Thus, no previous work has investigated age dependence for values of DKI metrics in anatomically defined areas using ROI-based parameter estimation in humans during adulthood. Regarding the magnitude of age-related changes, FA reportedly declines at a rate of approximately 3% per decade (28). The magnitude of change for MK mentioned above represents a decline of 1.4% and 2.1% per decade in WM and GM, respectively, if the decline were to be linear and start at 20 years.

Until now, reports on values of parameter estimates from DKI in the healthy brain have been scarce. Our values show a reasonable agreement with the limited number of areas that previously have been reported (Table 2). Standard deviations relative mean values were acceptable for MK, MD, and FA, 5–15% (Table 2), suggesting relatively high reliability. In the internal capsule, a pattern similar to that previously reported for DTI metrics was found (4, 20), with both MK and FA increasing stepwise from anteriorly in the ALIC to posteriorly in the PLIC, consistent with the strong positive correlation between these parameters (Fig. 3).

In DKI, inter-subject variability largely determines the within-group variability (29), and may also partly explain differences between studies. Substantial inter-subject variability of DTI parameters has been shown in several previous works, most of these aiming at studying the age dependence of DTI parameters (3, 4, 15). However, comparisons between studies are hampered by ROIs differing between studies, from including the entire structure to including only contiguous voxels with the highest values. For example, the doubling of FA in the genu of the corpus callosum from 0.47 ± 0.09 in (23) to 0.80 ± 0.04 in the present study, may be largely due to the ROI used for measurement, apart from age-related differences and inter- and within-scan as well as intersubject variability. It should be noted that regional variability of DTI values between publications may relate to using ROIs, voxel-based, tract-based, and manual tractography methods aside from acquisition parameters.

A limitation of the present study was that the data was obtained in 2 × 2 × 2 mm^3^ voxels. While this ensured accurate parameter estimates by minimizing the influence of partial volume effects, it also resulted in a suboptimal signal to noise ratio. The non-Gaussian distribution of the magnitude MRI signal at high *b*-values results in a bias in MK that varies with SNR (25). The globus pallidus (GP) has been suggested as a suitable area for testing whether the SNR is sufficient for DKI at 3T (1), with adequate SNR resulting in its MK value being similar to that of other GM regions. However, at low SNR, MK in the GP can become elevated relative other GM regions because the SNR induced bias is enhanced by the comparatively short T2 of the globus pallidus. MK in the GP and the putamen combined was 0.74 ± 0.1 (1), compared with 1.06 ± 0.08 in the GP and 0.67 ± 0.08 in the putamen reported in the present work. For comparison, in the thalamus MK was 0.93 ± 0.25 in (1), and 0.86 ± 0.07 in the present study. Hence, we cannot exclude that MK was positively biased in the globus pallidus in our study. However, we believe this bias to be less of a problem in white matter where inspections of the data showed acceptable signal to noise ratios even at high *b*-values. Finally, with regard to age dependency of MK and RK estimates, previous studies on DTI parameters (15, 30) showing such relationships have included large datasets, with the magnitude of the number of subjects included improving the statistical power, for example 202 subjects aged 5 to 30 years and 119 subjects aged 7 to 68 years, respectively. Our results failing to consistently show nonlinear age effects in WM and GM for any parameter, including FA and MD, indicate that the present study was underpowered as regards nonlinear effects of age dependency.

In conclusion, we measured DKI metrics in a large number of anatomically defined areas and found a two-fold increase in MK, accompanied by a four-fold increase in RK, when going from deep nuclei (i.e., the caudate head), to extremely directionally ordered WM in the splenium of the corpus callosum. MK and FA showed a strong positive correlation and RK and RD a strong negative correlation. Age dependence was found for MK, RK, MD, RD, and FA in some areas; linear age dependence was found for MK and RK in pooled WM and GM data, respectively. Estimates of MK in the internal capsule, corpus callosum and thalamus were in line with previous works. The range of values of parameter estimates overlapped with those reported for pathologies such as ischemic stroke, intracranial tumors, and neurodegerative disease.

## References

[1] Jensen J, Helpern JA (2010). MRI quantification of non-Gaussian water diffusion by kurtosis analysis. NMR Biomed.

[2] Cheung MM, Hui ES, Chan KC, Helpern JA, Qi L, Wu EX (2009). Does diffusion kurtosis imaging lead to better neural tissue characterization? A rodent brain maturation study. Neuroimage.

[3] Lebel C, Walker L, Leemans A, Phillips L, Beaulieu C (2008). Microstructural maturation of the human brain from childhood to adulthood. Neuroimage.

[4] Schneiderman JS, Buchsbaum MS, Haznedar MM (2007). Diffusion tensor anisotropy in adolescents and adults. Neuropsychobiology.

[5] Qian W, Zhang Z, Wu E

[6] Helpern JA, Adisetiyo V, Falangola MF (2011). Preliminary evidence of altered gray and white matter microstructural development in the frontal lobe of adolescents with attention-deficit hyperactivity disorder: a diffusional kurtosis imaging study. J Magn Reson Imaging.

[7] Lu H, Jensen JH, Ramani A, Helpern JA (2006). Three-dimensional characterization of non-Gaussian water diffusion in humans using diffusion kurtosis imaging. NMR Biomed.

[8] Falangola MF, Jensen JH, Babb JS (2008). Age-related non-Gaussian diffusion patterns in the prefrontal brain. J Magn Reson Imaging.

[9] Good CD, Johnsrude IS, Ashburner J, Henson RN, Friston KJ, Frackowiak RS (2001). A voxel-based morphometric study of ageing in 465 normal adult human brains. Neuroimage.

[10] Sowell ER, Thompson PM, Toga AW (2004). Mapping changes in the human cortex throughout the span of life. Neuroscientist.

[11] Moseley M (2002). Diffusion tensor imaging and aging - a review. NMR Biomed.

[12] Camara E, Bodammer N, Rodriguez-Fornells A, Tempelmann C (2007). Age-related water diffusion changes in human brain: a voxel-based approach. Neuroimage.

[13] Kochunov P, Thompson PM, Lancaster JL (2007). Relationship between white matter fractional anisotropy and other indices of cerebral health in normal aging: tract-based spatial statistics study of aging. Neuroimage.

[14] Sullivan EV, Pfefferbaum A (2007). Neuroradiological characterization of normal adult ageing. Br J Radiol.

[15] Lebel C, Gee M, Camicioli R, Wieler M, Martin W, Beaulieu C (2011). Diffusion tensor imaging of white matter tract evolution over the lifespan. Neuroimage.

[16] Lu H, Jensen JH, Hu C

[17] Poot DH, den Dekker AJ, Achten E, Verhoye M, Sijbers J (2010). Optimal experimental design for diffusion kurtosis imaging. IEEE Trans Med Imaging.

[18] Jensen JH, Helpern JA, Ramani A, Lu H, Kaczynski K (2005). Diffusional kurtosis imaging: the quantification of non-gaussian water diffusion by means of magnetic resonance imaging. Magn Reson Med.

[19] Tabesh A, Jensen JH, Ardekani BA, Helpern JA (2011). Estimation of tensors and tensor-derived measures in diffusional kurtosis imaging. Magn Reson Med.

[20] Kawaguchi H, Obata T, Ota M (2010). Regional heterogeneity and age-related change in sub-regions of internal capsule evaluated by diffusion tensor imaging. Brain Res.

[21] Wang JJ, Lin WY, Lu CS (2011). Parkinson disease: diagnostic utility of diffusion kurtosis imaging. Radiology.

[22] Raab P, Hattingen E, Franz K, Zanella FE, Lanfermann H (2010). Cerebral gliomas: diffusional kurtosis imaging analysis of microstructural differences. Radiology.

[23] Van Cauter S, Veraart J, Sijbers J (2012). Gliomas: diffusion kurtosis MR imaging in grading. Radiology.

[24] Lätt J, van Westen D, Nilsson M

[25] Fieremans E, Jensen JH, Helpern JA (2011). White matter characterization with diffusional kurtosis imaging. Neuroimage.

[26] Hasan KM, Sankar A, Halphen C (2007). Development and organization of the human brain tissue compartments across the lifespan using diffusion tensor imaging. Neuroreport.

[27] Hasan KM, Halphen C, Boska MD, Narayana PA (2008). Diffusion tensor metrics, T2 relaxation, and volumetry of the naturally aging human caudate nuclei in healthy young and middle-aged adults: possible implications for the neurobiology of human brain aging and disease. Magn Reson Med.

[28] Grieve SM, Williams LM, Paul RH, Clark CR, Gordon E (2007). Cognitive aging, executive function, and fractional anisotropy: a diffusion tensor MR imaging study. AJNR Am J Neuroradiol.

[29] Szczepankiewicz F, Leemans A, Sundgren PC

[30] Zhu T, Hu R, Qiu X (2011). Quantification of accuracy and precision of multi-center DTI measurements: a diffusion phantom and human brain study. Neuroimage.

